# Analysis of predictive factors for R0 resection and immediate bleeding of cold snare polypectomy in colonoscopy

**DOI:** 10.1371/journal.pone.0213281

**Published:** 2019-03-01

**Authors:** Tomonori Aoki, Shuntaro Yoshida, Hiroyuki Abe, Satoshi Ono, Ayako Nakada, Yumiko Ota, Akiko Narita, Takeshi Yoshikawa, Hiroto Kinoshita, Yosuke Tsuji, Atsuo Yamada, Mitsuhiro Fujishiro, Yoshihiro Hirata, Masashi Fukayama, Kazuhiko Koike

**Affiliations:** 1 Department of Gastroenterology, Graduate School of Medicine, The University of Tokyo, Tokyo, Japan; 2 Department of Endoscopy and Endoscopic Surgery, The University of Tokyo, Tokyo, Japan; 3 Department of Pathology, Graduate School of Medicine, The University of Tokyo, Tokyo, Japan; University of Munich, GERMANY

## Abstract

**Background:**

Factors associated with efficacy and safety of cold snare polypectomy (CSP) are not well established. The aim is to elucidate the predictors of R0 resection and immediate bleeding of CSP.

**Methods:**

We retrospectively reviewed a database of patients who underwent CSP for subcentimetric polyps at the University of Tokyo Hospital in Japan. Using the data regarding the characteristics of patients and polyps, such as location, size, and macroscopic appearance; use of narrow band imaging with magnification (NBI-M); and endoscopists’ experience, we revealed the predictive factors associated with R0 resection and immediate post-CSP bleeding by univariate and multivariate analyses.

**Results:**

In total, 399 polyps, in 200 patients without antithrombotics, were removed. Failure of tissue retrieval was noted in 4% of resected lesions. There was no intramucosal carcinoma observed. The overall rate of R0 resection was 46%. Multivariate analysis elucidated that the observation of the polyp with NBI-M was an independent predictor associated with R0 resection (odds ratio [OR] 1.90; p = 0.024). Although immediate post-CSP bleeding occurred in 19 polyps (4.8%), no delayed bleeding or perforation was observed. Multivariate analysis revealed protruded lesion as an independent risk factor for immediate bleeding (OR 3.54; p = 0.018).

**Conclusions:**

A higher rate of R0 resection with CSP can be achieved by performing colonoscopy with NBI-M, than with white-light imaging. Macroscopic protruding appearance of a polyp is a risk factor for immediate bleeding.

## Introduction

Colorectal cancer (CRC) is a fatal cancer that ranks fourth around the world, and second in Japan, in mortality among all malignant disorders.[1,2] Colonoscopy is associated with a reduced incidence and mortality of CRC, and endoscopic polypectomy at the time of colonoscopy has become effective in interrupting the progression of the adenoma-carcinoma sequence, consequently preventing death from CRC.[3–6]

As for endoscopic polypectomy, a R0 resection of polyps should be performed, since incomplete resection is considered to account for 19% to 30.8% of interval CRC.[7–9] In addition, polypectomy carries a definite risk of complications, such as bleeding or perforation.[3,10] Thus, it is desirable for the therapeutic procedure of polypectomy to provide the high R0 resection rate and safety.

Previous studies have revealed that most polypectomies are performed for subcentimetric (< 10 mm) lesions, which represent over 80% of all polyps, and recently, the high efficacy and safety of cold polypectomy without electrocautery current for subcentimetric polyps has been reported.[11] In general, cold polypectomy consists of cold forceps polypectomy (CFP) and cold snare polypectomy (CSP). CSP has been determined to be superior to CFP in the R0 resection rate of small colorectal polyps.[12,13] Additionally, there are few complications, such as delayed bleeding or perforation, after CSP.[11,14–16] Thus, CSP is a safe and reliable technique for the treatment of diminutive and small colorectal polyps.

Although high R0 resection rates were achieved in prospective trials in which the histologic eradication was assessed by biopsy samples from the edge of resected polys or by endoscopic mucosal resection of the polypectomy site, only 59% of the neoplastic lesions removed by CSP were classified as horizontal margins (HMs) 0 (*i*.*e*., no tumor identified at the lateral margin) in the conventional histologic evaluation method of clinical practice.[12,13,17,18] It also should be noted that several studies reported immediate bleeding following lesion removal, requiring endoscopic hemostasis.[11,18]

Therefore, the knowledge of the factors that lead to incomplete resection and complications is significant; however, the evaluation of factors associated with efficacy and safety of CSP is deficit. Accordingly, we conducted a retrospective study to elucidate the predictive factors of R0 resection and complications of CSP for subcentimetric lesions.

## Materials and methods

### Study design, setting, and participants

We retrospectively identified patients who underwent colorectal CSP for a subcentimetric polyp at the University of Tokyo Hospital in Japan, from December 2013 to November 2014. Data was collected from an endoscopic database, which is a searchable collection of records into which endoscopists prospectively add data after the procedure. CSP was not performed for lesions with suspected carcinomas, based on endoscopic assessments. CSP was not performed without the cessation of antithrombotic agents. This study complied with the Declaration of Helsinki. The design was approved by the ethics committee of The University of Tokyo (approval number 2058). This study was a retrospective observational study, and informed consent to participate was carried out by the opt-out method of our hospital website. Patient information was anonymized and deidentified before analysis.

### Procedures

**Fig 1:**
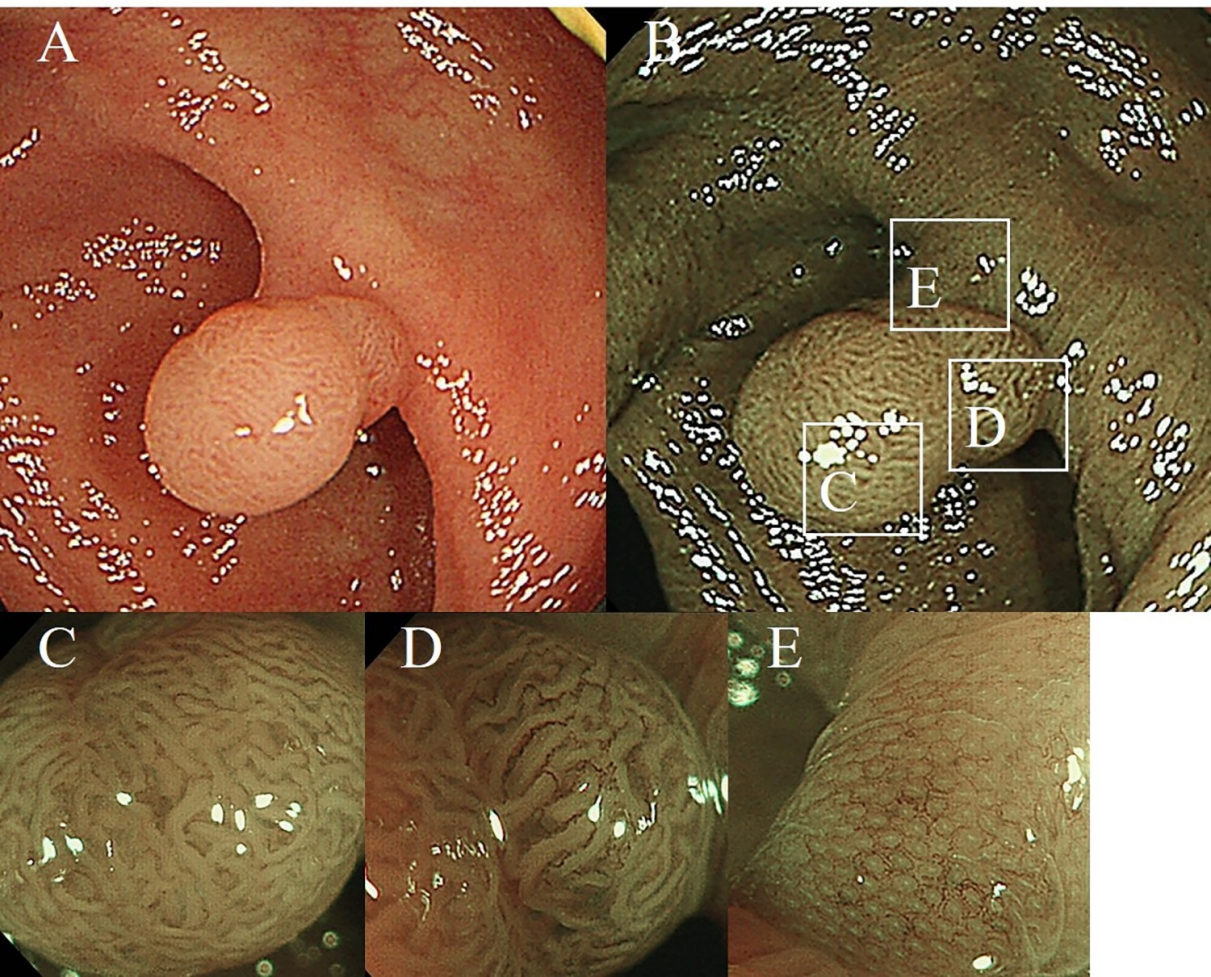
Qualitative and quantitative evaluation of colonic polyp with WLI (A), NBI (B) and NBI-M (C, D, and E).

Colonoscopy was performed using electronic video endoscopes (type PCF-240I, PCF-Q260AI, or PCF-PQ260L; Olympus Optical, Tokyo, Japan) or a magnifying electronic video endoscope (PCF-Q260AZI; Olympus Optical, Tokyo, Japan). The prediction of polyp histopathology was performed with or without magnification to determine the indication for CSP.[19,20] We also evaluated the lateral expanse of the lesion with white light imaging (WLI) and NBI **(Fig 1)**. Bowel preparation was performed using a 2-L polyethylene glycol electrolyte lavage solution. Additionally, CSP was performed using a single-use oval snare, with a loop width of 13 mm (Rotatable snare; Boston Scientific, Natick, MA, USA), without electrocautery.[21] Subsequently, the transected polyp was retrieved through the colonoscopic suction channel, into the gauze placed over the suction port. To maintain a non-neoplastic mucosal margin for polypectomy, we captured the polyp with 1 mm to a few mm of surrounding normal tissue located around the lesion. Colorectal polyps up to 9 mm, without suspected carcinomas, were removed by CSP if the polyps could be easily captured with the surrounding normal tissue. For polyps that we could not technically resect using the extended resection method, such as polyps at the strong bending or fixed location, we performed CFP or endoscopic mucosal resection (EMR). In our institution, hot snare polypectomy (HSP) was not routinely performed. Polyps of 10mm or larger, or suspected carcinomas were resected by EMR or endoscopic submucosal dissection. Following CSP, the polypectomy ulcer was rinsed by forceful irrigation with water, and to confirm the endoscopic complete resection of the polyp. Hemostatic clipping was carried out during the procedure for immediate bleeding, and hemostasis was obtained in all patients at the end of colonoscopy. The retrieved specimens were immersed in 20% formalin without pinning on a plate. After fixation, the specimens were embedded in paraffin blocks. Single largest section was prepared from each specimen and then examined using standard hematoxylin and eosin staining. Thereafter, the success of histologic eradication was judged by performing a histologic diagnosis, determined according to the criteria from the World Health Organization.

All patients who underwent CSP visited our hospital two weeks after procedure to be informed of the histopathological results from the polyps that were removed. At the same time, they were interviewed for post-CSP complications, including bleeding and perforation. For patients who complained of equivocal symptoms, the presence of progressive anemia was investigated by performing a blood test, as necessary.

### Parameters

All required variables (i.e., characteristics of patients, characteristics of polyps such as location, size, and macroscopic appearance); use of NBI-M (i.e., narrow-band imaging with magnification); and details on the endoscopists’ experience were collected from a recorded colonoscopic database, into which endoscopists prospectively input data following the completion of endoscopies. The polyp location was categorized as either right side (i.e., cecum, ascending colon, or transverse colon) or left side (i.e., descending colon, sigmoid colon, or rectum). The size of each polyp was determined using a 13-mm snare for CSP, or the opening width of the biopsy forceps (FB-240U; Olympus Optical, Tokyo, Japan). The polyp size was classified as either diminutive (i.e., 1 mm to 5 mm) or small (i.e., 6 mm to 9 mm).[22] The macroscopic appearance was classified as either superficial or protruded, according to the Paris classification.[23] Use of NBI-M was identified by the colonoscope information and endoscopic images. CSP was performed by 21 endoscopists, including eight experts with experience of ≥ 3,000 colonoscopies, and 13 non-experts.[24]

### Outcome measures

We assessed the R0 resection or CSP-related complications including post-CSP bleeding and perforation. R0 resection was defined as en bloc resection with histologically assessed clear margins[25]. One experienced pathologist, who was blinded to the endoscopic findings, re-evaluated the horizontal margins of all of the polyp specimens in this study. For the purpose of this study, post-CSP bleeding includes immediate bleeding and delayed bleeding.[26] Immediate bleeding that required hemostatic clipping was defined as spurting or oozing that continued after resection for more than 30 seconds, while delayed bleeding was defined as bleeding within two weeks after CSP, requiring endoscopic intervention.

### Statistics

Predictive factors for R0 resection and post-CSP bleeding were evaluated by univariate analysis using Pearson’s Chi-squared test or Fisher’s exact test as appropriate. Logistic regression analysis was used to estimate crude and adjusted odds ratios (OR) and 95% confidence intervals (CI). As for post-CSP bleeding, 19 bleeding events allowed for the analysis of two variables at most in multiple analyses, and then we entered only variables with a univariate significance of P < 0.2 into the multivariate analysis.[27] P-values < 0.05 were considered significant. We did not perform sample estimation because our study was strongly based on a descriptive and exploratory design. All data were statistically analyzed using STATA version 13 software (StataCorp, College Station, TX, USA).

## Results

### Patient and polyp characteristics

During the study period, 2042 subcentimetric lesions were resected (CSP, 399 lesions; CFP, 410 lesions; EMR, 1233 lesions). CSP was attempted in 200 patients (male, 72%; mean age, 65 y; range, 32–89 y) **(Table 1)**. CSP procedures in 77 (39%) of the patients were performed by non-experts, while experts performed CSP in 123 (61%) of the patients. No one was undergoing antithrombotic therapy at the time of procedure. The major indication for colonoscopy was surveillance (i.e., post-polypectomy or post-colorectal cancer resection). In total, 399 subcentimetric lesions were resected by CSP **(Table 2)**. Of these, 182 (46%) were located in the left side of colon, and 355 (89%) were diminutive. Two hundred and twenty-three (56%) were superficial/elevated (0-IIa), 167 (42%) were protruded/sessile (0-Is), and eight (2%) were protruded/pedunculated (0-Ip). NBI-M was performed for 75 (19%) lesions. Retrieval was unsuccessful in 15 (4%) of resected lesions. Histologically, the majority of polyps evaluated were adenomatous polyps (85%), and there was no intramucosal carcinoma.

**Table 1:** Baseline characteristics of patients (n = 200).

Characteristic	Value
Mean age, years (± SD)	65 ± 11
Age ≥65 years, n (%)	140 (70)
Sex, Male/Female, n (%)	143 (72)/57 (28)
Endoscopist, non-expert/expert, n (%)	77 (39)/123 (61)
Antithrombotic drugs, n (%)	0 (0)
Procedure times, min. (± SD)	
Insertion	10 ± 7
Withdrawal	26 ± 14
Indications, n (%)	
Screening	52 (26)
Surveillance	100 (50)
Bleeding/anemia	19 (10)
Pain	4 (2)
Diarrhea	2 (1)
Other	23 (12)

Values presented with a plus/minus sign are means ± standard deviation.

**Table 2:** Baseline characteristics of polyps (n = 399).

Characteristic	Value
Location, Left/Right side of colon, n (%)	182 (46)/216 (54)
Size, Small/Diminutive, n (%)	44 (11)/355 (89)
Shape, Flat/Sessile/Pedunculated, n (%)	223 (56)/167 (42)/8 (2)
NBI-M, Yes/No, n (%)	75 (19)/324 (81)
Histology, n (%)	
Adenoma	340 (85)
Tubular adenoma	333 (83)
Sessile serrated adenoma / polyp	4 (1)
Tubulovillous adenoma	2 (1)
Serrated adenoma	1 (0.2)
Hyperplastic polyp	35 (9)
Other polyps, mucosal tag, or inflammation	9 (2)
Tissue retrieval failure	15 (4)

Abbreviations: NBI-M, narrow-band imaging with magnification.

### The rate and predictive factors of R0 resection

Among 377 polyps (i.e., 340 adenomatous polyps, 35 hyperplastic polyps, and 2 juvenile polyps), the overall rate of R0 resection was 46% (**Table 3**). We excluded lesions that were either not retrieved (n = 15, 4%) or diagnosed as mucosal tag or inflammation (n = 7, 2%). The predictive factors for R0 resection are shown in **Table 4**. Univariate analysis revealed the observation of the polyp with NBI-M (OR, 1.83; 95% CI, 1.09–3.09; p = 0.021) as the significant predictor for R0 resection, and multivariate regression analysis elucidated NBI-M (OR, 1.90; 95% CI, 1.09–3.32; p = 0.024) to be the independent predictor for R0 resection.

**Table 3:** Efficacy and safety of CSP.

Outcome	Value
R0 resection, n (%)*	174 (46)
Complications, n (%)**	
Immediate bleeding	16 (8.0) ^†^
Delayed bleeding	0 (0)
Perforation	0 (0)

*histologically re-evaluated specimens other than mucosal tag or inflammation (n = 377)

**all patients (n = 200)

^†^19 polyps of 16 (8%) patients

Abbreviations: CSP, cold snare polypectomy.

**Table 4:** Predictive factors for R0 resection (n = 377).

	R0 resection (n = 174)	Univariate analysis		Multivariate analysis	
		Crude OR (95% CI)	P value	Adjusted OR (95% CI)	P value
Age, n (%)					
≥65	93 (42)	0.69 (0.46–1.04)	0.074	0.69 (0.45–1.05)	0.085
<65	81 (52)				
Sex, n (%)					
Male	129 (46)	0.96 (0.60–1.53)	0.870	0.90 (0.56–1.45)	0.674
Female	45 (47)				
Location, n (%)					
Left	88 (51)	1.47 (0.98–2.21)	0.066	1.35 (0.88–2.05)	0.165
Right	86 (42)				
Size, n (%)					
Small	21 (49)	1.13 (0.60–2.13)	0.708	1.12 (0.58–2.14)	0.736
Diminutive	153 (46)				
Shape, n (%)					
Protruded	75 (45)	0.95 (0.63–1.43)	0.810	1.04 (0.68–1.59)	0.857
Superficial	99 (47)				
Endoscopist, n (%)					
Non-expert	60 (43)	0.83 (0.54–1.26)	0.373	1.02 (0.65–1.59)	0.945
Expert	114 (48)				
NBI-M, n (%)					
Yes	42 (58)	1.83 (1.09–3.09)	**0.021**	1.90 (1.09–3.32)	**0.024**
No	132 (43)				

Abbreviations: OR, odds ratio; CI, confidence interval; NBI-M, narrow-band imaging with magnification.

### The rate and risk factors of post-CSP bleeding

Although immediate post-CSP bleeding requiring endoscopic hemostasis occurred in 19 polyps of 16 (8%) patients, no delayed bleeding requiring endoscopic intervention after CSP or perforation was observed (**Table 3**). The risk factors for immediate bleeding are shown in **Table 5**. Univariate analysis revealed protruded lesion (OR, 3.77; 95% CI, 1.33–10.7; p = 0.013) as the significant risk factor for immediate bleeding, and multivariate regression analysis elucidated protruded lesion (OR, 3.54; 95% CI, 1.24–10.1; p = 0.018) as the independent risk factor for immediate bleeding.

**Table 5:** Risk factors for immediate post-CSP bleeding (n = 399).

	Immediate bleeding (n = 19)	Univariate analysis		Multivariate analysis	
		Crude OR (95% CI)	P value	Adjusted OR (95% CI)	P value
Age, n (%)					
≥65	9 (3.8)	0.60 (0.24–1.51)	0.278		
<65	10 (6.2)				
Sex, n (%)					
Male	15 (5.0)	1.27 (0.41–3.91)	0.680		
Female	4 (4.0)				
Location, n (%)					
Left	8 (4.4)	0.86 (0.34–2.18)	0.745		
Right	11 (5.1)				
Size, n (%)					
Small	3 (6.8)	1.55 (0.43–5.55)	0.500		
Diminutive	16 (4.5)				
Shape, n (%)					
Protruded	14 (8.0)	3.77 (1.33–10.7)	**0.013**	3.54 (1.24–10.1)	**0.018**
Superficial	5 (2.2)				
Endoscopist, n (%)					
Non-expert	10 (6.7)	1.90 (0.76–4.80)	0.172	1.63 (0.64–4.17)	0.307
Expert	9 (3.6)				
NBI-M, n (%)					
Yes	3 (4.0)	0.80 (0.23–2.83)	0.731		
No	16 (4.9)				

Abbreviations: CSP, cold snare polypectomy; OR, odds ratio; CI, confidence interval; NBI-M, narrow-band imaging with magnification.

## Discussion

In this retrospective study, we focused on the efficacy and safety of CSP and those associated factors. Our results show the low rate of retrieval failure, the absence of intramucosal carcinoma and the low rate of complications, which indicated the high efficacy and safety of CSP. In addition, we reveal that CSP with NBI-M was a positive predictor for R0 resection, while protruded lesion was the risk factor for immediate post-CSP bleeding.

The rate of R0 resection was lower in this study (i.e., 46%) than in previous prospective studies (i.e., over 90%).[12,13,28] This discrepancy may be due to the different methods for evaluating R0 resection, or the different types of snares used. Furthermore, in prior studies, samples collected using two or more additional biopsies after CSP were used for evaluation[13], or excised specimens were mounted with pins on Styrofoam plates and fixed in 10% formalin to evaluate lateral margins.[28] In this study, however, we evaluated the R0 resection using only the CSP specimens themselves, without any additional methods of fixation. It is convenient for pathologists to identify the edge of tissues if the polyps were resected with electrocautery. As for CSP, it tends to be more difficult to assess the horizontal margins in order to estimate. Indeed, a retrospective study involving the similar method of histologic evaluation from Japan reported that the negative horizontal margin rate was 59%, which was comparable with our results.[18]

Another reason for the low R0 resection rate may be the fact that the snare, which we adapted, was not specifically designed for CSP. In actuality, the dedicated cold snare (Exacto cold snare, US Endoscopy, Mentor, OH, USA) showed the advantage in complete resection rate in previous studies.[12,28] The snare, which we adapted, allowed for the method of polypectomy with or without electrocautery current to be successful, and appears to be more suitable for use in the routine colonoscopy procedure from the clinical and economic perspectives. Indeed, using the same snare for CSP, we performed polypectomy with electrocautery current in the case of 41 (21%) patients in this CSP study.

Because incomplete histologic resection of neoplastic polyps may contribute to the development of CRC after colonoscopy, it is important to keep the high complete histologic resection rate in polypectomy. Although a recent study[29] suggested that polyp lateral margins could not be useful in assessing the completeness of resection, it still seems meaningful to achieve negative histopathological margin of polyps. Incomplete resection cases existed when failed to obtain negative horizontal margin in that study, actually we experience such cases in clinical practice. With respect to completeness of resection, both CSP and CFP fail to R0 resection to some degree, and CFP is known to be inadequate for diminutive polyps because of the low R0 resection rate (i.e., 39%).[30] Lee et al. reported that the rate of recurrence after CFP was 17% during a median follow-up period of 59.7 months (Interquartile range [IQR], 41.1–75.6 months) in a retrospective study, whereas it remains unknown about recurrence after CSP.[31] As such, the rate of recurrence after CSP should be evaluated in a prospective study.

We clarify CSP with NBI-M as a new predictor for R0 resection. This study's results indicate that NBI-M may be a promising tool not only for qualitative diagnosis to prevent from removing subcentimetric carcinomas in CSP as previously reported, but also for the precise evaluation of a lateral neoplastic extent in order to achieve R0 resection.[32] Inconsistent with previous studies, polyp size was not the associated factor for R0 resection in our study.

The incidence of immediate bleeding requiring endoscopic hemostasis in the present study was low (i.e., 4%, per-polyp), and was consistent with previous studies demonstrating 2% to 3%, per-polyp.[11,18] Additionally, we found that protruded lesion was the risk factor for immediate bleeding, although no studies have proposed this previously as a risk factor. Repici et al. reported that antiplatelet agents and larger polyp size (i.e., 6 mm to 9 mm) were independent predictors of bleeding, but the study included CFP (63%) in addition to CSP (37%).[11] Takeuchi et al. also proposed that larger polyp size (i.e., 6 mm to 9 mm) was the predictor of bleeding, but there were not enough bleeding cases (n = 8) included in the study to reveal this relationship.[18] Thus, the bleeding risks of CSP are not yet concluded. However, thinking of the result of a prospective study, which showed that intraprocedural bleeding was significantly more frequent in CSP group than that in a HSP group, special attention should be paid to bleeding risks such as protruded polyps and larger polyps on CSP.[16] It is unclear why protruded lesions carry the higher risk than superficial lesions, but it is possible that higher (i.e., larger-volume) polyps have the supply of thicker blood vessels than lower (i.e., smaller-volume) polyps, which may lead to bleeding after CSP without spontaneously resolving.

Our study had some limitations. In addition to the relatively small sample size, this was a retrospective single-center study without long-term outcomes consideration of CSP. Because of the retrospective design, the exact times after CSP could not be confirmed, although we intended to wait for 30 seconds before hemostatic clipping. In addition, although the extended resection method seemed important for R0 resection, the extended resection method could not be precisely confirmed by photographs or videos. Therefore, we could not evaluate the association between the extended resection method and R0 resection. Furthermore, different from several randomized controlled studies[12,13], we could not evaluate the surrounding mucosa with biopsy or EMR after CSP for assessing the residual polyp tissue.

Despite these limitations, however, the study also had several strengths. First, this study was performed by 21 endoscopists, so results can be generalized in clinical practice. Second, one experienced pathologist re-evaluated the horizontal margins of all of the polyp specimens for this study, which provided unified estimation for R0 resection.

In conclusion, CSP has a high level of safety, and could be considered as the reasonable procedure for subcentimetric polys removal. A higher rate of R0 resection with CSP can be achieved by colonoscopy with NBI-M than with white-light imaging. Following CSP for protruded lesions, we should pay attention to post-CSP bleeding, especially immediate bleeding.

## Abbreviations

CSP: cold snare polypectomy
CFP: cold forceps polypectomy
EMR: endoscopic mucosal resection
HSP: hot snare polypectomy
OR: odds ratio
CI: confidence interval
HMs: horizontal margins
NBI-M: narrow-band imaging with magnification
WLI: white light imaging
SD: standard deviation
